# First steps in PROMs and PREMs collection in Wales as part of the prudent and value-based healthcare agenda

**DOI:** 10.1007/s11136-020-02711-2

**Published:** 2020-11-29

**Authors:** Kathleen Withers, Robert Palmer, Sally Lewis, Grace Carolan-Rees

**Affiliations:** 1 Cedar Healthcare Technology Research Centre, Cedar, Cardiff and Vale University Health Board, Cardiff Medicentre, Heath Park, Cardiff, CF14 4UJ UK; 2 Value Based & Prudent Healthcare, Mamhilad House, Mamhilad Park Estate, Pontypool, NP4 0HZ UK

**Keywords:** Shared decision making, Health-related quality-of-life, Value-based healthcare, Patient engagement, Patient-reported outcomes, PROMS

## Abstract

**Purpose:**

Patients are experts in their own health and should be treated as equal partners in their care. Patient-reported outcome measures (PROMs) are an effective way of gathering patient feedback and can facilitate effectiveness and cost-effectiveness analysis to improve decision making and service improvement. The PROMs, PREMs & Effectiveness Programme was initiated in 2016 and aimed to develop an electronic platform to facilitate collection of PROMs and Patient-reported experience measures (PREMs) from secondary care patients across Wales.

**Methods:**

We worked with all Health Boards in Wales, the NHS Wales Informatics Service (NWIS), and Cedar (a healthcare technology research centre) to identify and meet technical requirements to develop a platform which is fit for purpose. Patient groups were included throughout the development to gather feedback and for extensive testing. Clinical teams helped identify the most appropriate tools, with licences, translations and electronic formatting issues being managed centrally.

**Results:**

The developed platform is integrated with patient administration systems minimising the need for manual input, with processes in place to allow automatic collection triggers according to nationally agreed schedules. We have over 30 nationally agreed PROMs ‘pathways’ with over 110,000 PROMs collected to date. Responses are fed back to clinicians via the electronic patient record and to each health board via feeds to the national data warehouse, making data easily accessible to different teams, maximising use and application.

**Discussion:**

The national platform has provided a co-ordinated approach to PROMs collection in Wales, offering an effective means of communicating with patients outside the traditional clinic visit.

## Introduction

Clinicians worldwide are experts at treating diseases and conditions, with new advances leading to continued changes in healthcare provision. While the main purpose of any healthcare system is to promote, restore and/or maintain health, arguably improving health ultimately aims to enhance health-related quality of life (HRQoL) [1]. Improved health may be achieved by a range of methods including better disease prevention, and improving curative, supportive and palliative care. While objective measures such as disease, survival and infection rates (for example) can be readily assessed by clinical teams, quality-of-life measures such as subjective wellbeing can only be assessed by patients themselves [2]. In order to identify what matters most to patients in relation to their health, we need to identify the symptoms, impacts and outcomes of greatest importance to them. This can help us support improved HRQoL which specifically refers to the health aspects of quality of life, generally considered to reflect the impact of disease and treatment on disability and daily functioning [3].We should listen to patients to understand the experience of care that they have received so we can improve provision wherever possible. Increasingly, patients are being asked to provide their views on their health and healthcare experiences using questionnaires or tools called patient-reported outcome measures (PROMs) and patient-reported experience measures (PREMs). PROMs measure patient symptoms and health-related quality of life from the patient’s perspective, often before and after (and sometimes during) treatment to identify any related changes [4]. PROMs were originally used in research but have been adopted into more widespread practice, particularly notable perhaps in England and Sweden. In Sweden, the National Quality Registers which collect patient-level data on treatment and outcomes were collecting PROMs by 2002 [5]. Swedish Quality Registers are now obliged to include PROMs for high-level certification and many report examples of how they are used for quality improvement initiatives such as shared decision making [6]. National PROMs collection started in England in 2009, initially focussing on four clinical areas: hip replacement, knee replacement, varicose vein surgery, and hernia surgery, and aims to enable change by identifying good practice, financially penalising poor providers and facilitating transparency [7] (PROMs collection in varicose vein and hernia surgery was discontinued in October 2017). As well as facilitating improved care on an individual patient level by providing an individualised service based on patient-reported data [8], co-ordinated PROMs collection can identify areas with relative good or poor outcomes through benchmarking, which can facilitate improved clinical performances [9]. It can also provide collated quality-of-life datasets to support decision makers such as funders by allowing healthcare providers to assess the effectiveness and cost effectiveness of care. However, despite a few large-scale initiatives, it has been suggested that PROMs collection is often sub-optimal and fragmented with limited co-ordination [10].

As their name suggests, PREMs measure patients’ experiences of care and may include questions about how well information was explained to them, whether they had opportunities to ask questions, and whether staff was polite [11]. PREMs can also be used to improve services by identifying areas with good and poor practice to drive service improvement. Research has suggested that there may be a direct relationship between patients’ experiences and their outcomes, particularly in relation to trust and the level of communication with their clinician [12].

### Value-based healthcare

PROMs are recognised as having a role in the rapidly growing worldwide movement of value-based healthcare (VBHC), which aims to maximise the value of care provided for patients within available resources. It has been defined as the health outcomes achieved per dollar of cost [13]. Recent efforts have been made to further define “Value” in VBHC [14], with the suggestion that access to care is important, and allocation of resource being a factor in ensuring that those being treated are those who will benefit most [15]. VBHC is intrinsically linked to PROMs as the value of healthcare should always focus on the patient and be measured by outcomes and not volume of services delivered [16]. A recent assessment found alignment with VBHC approaches in countries including Sweden, England, US, South Korea and Colombia [17]. The co-ordinated collection of PROMs facilitates the systematic involvement of patient feedback into programmes which aim to improve the cost effectiveness and quality of healthcare provided. Their use can allow us to identify those with the greatest need and to reduce inappropriate variation using evidence-based approaches [18]. Doing only what is needed at the right time, and being able to assess and compare different providers, treatments and regimes can not only provide better outcomes for patients through shared learning but also ensure that these can be delivered in the most cost efficient way [9].

Wales is one of the numerous countries worldwide in which VBHC is being seen as a way of delivering healthcare in a prudent way to secure sustainable services [19]. It relies on data to drive improvements in clinical outcomes for patients and strives to provide tailored care for each individual [4]. The availability of high-quality outcomes data can allow healthcare providers to identify and disinvest in low-value interventions and focus their resources more efficiently. The implementation of VBHC in Wales started at Aneurin Bevan University Health Board (ABUHB) in 2015 in response to the Welsh Government’s (WG) calls to meet the challenges of rising costs and increased demands while continuing to improve quality [20]. The focus on outcomes by the ABUHB programme has been recognised as an international example of how VBHC can be put into practice [20]. A recent annual report by the Chief Medical Officer in Wales [19] highlights its application across Wales, and the importance of co-production to involve patients as equal partners via the use of tools such as PROMs and PREMs. This requires the robust collection and reporting of outcome data.

## Purpose

In NHS Wales, PROMs and PREMs have been collected for a number of years, but this has primarily been driven via small local initiatives with a restricted approach to co-ordinated collection. This has reduced opportunities for shared learning from outcomes data. This limited uncoordinated collection has also restricted our ability to more broadly invite all of our patients to tell us about their experiences and outcomes of care. In order to facilitate standardised collection across Wales, the PROMs, PREMs and Effectiveness Programme (PPEP) was initiated [21]. This ambitious programme of work aimed to develop and roll out an electronic platform to collect PROMs and PREMs from all secondary care patients across Wales using a co-ordinated, top-down approach. As well as supporting the principles of co-production to help involve patients more in decisions about their care, unified collection and linkage of patient outcome data with clinical and administrative data have huge potential in supporting the VBHC agenda [22].

## Method

In early 2016, a successful grant application was made to the WG’s efficiency through technology fund to develop an electronic PROMs and PREMs data collection platform. Here, we provide a brief overview of the groups involved, methods used for tool selection and system integration.

To facilitate and support the development and roll out of the platform, a core team of staff was brought together under three primary work streams.

### Technical work stream

Based at the NHS Wales Informatics Service (NWIS), this group is responsible for the technical development, roll out and support of the electronic platform itself.

### Implementation & engagement work stream

This team comprises of two PPEP Programme managers and a Project Manager. Their role includes engagement with clinical groups across Wales to promote the PPEP and facilitate the collection of PROMs and PREMs on the system. This includes supporting change management within the health boards (HBs), ascertaining how collection will work best in different clinical and geographical areas, and identifying how to overcome obstacles to implementation.

### Analytical work stream

Based within Cedar, a healthcare technology research centre, this team focuses on data analysis and report writing while also working on PROM tool identification, licensing and Welsh language translations.

### Clinical leads

In addition to the three teams, national and local clinical leads were originally enrolled to facilitate engagement with clinical colleagues.

As the main driver for this work is to put patients at the centre of care, patients and patient groups were consulted to gather feedback on key aspects of the work.

### PROMs and PREMs selection

**Fig. 1:**
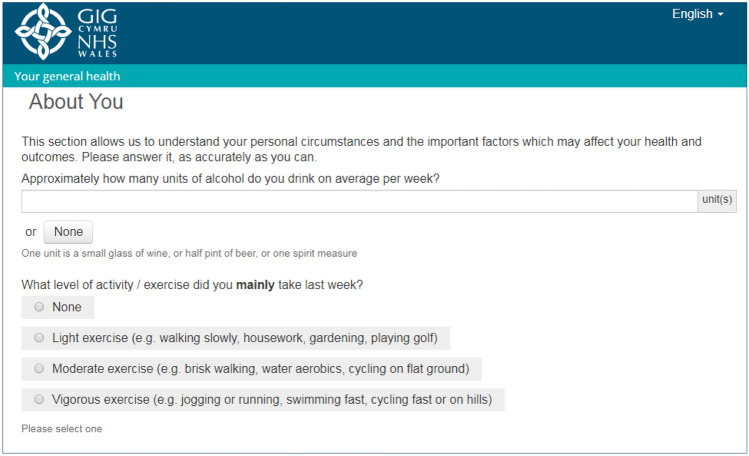
A screenshot of one of the “About You” questions on the national platform, to illustrate the patients view

Working with all local HBs and trusts in Wales, and supported by WG, the PPEP team sets out to identify requirements for a data collection platform including technical and practical drivers. It was decided that the system would collect a generic set of questions, supported, where agreed by condition-specific tools. The 5-level version of the EQ-5D (EQ-5D-5L) [23] was chosen as an appropriate generic PROM to support between-group/condition comparisons and health economic analysis due to its advantages over the 3L version [24]. The work, productivity and activity index (WPAI) [25] was also chosen to identify health-related reduction in work productivity. This short instrument was primarily included to help assess the impact of ill health and symptom severity on work and activities, and in assessing the wider economic impact of some conditions/treatments. Other “About You” questions were agreed to provide information on aspects of life, covering weight, height, exercise levels, alcohol intake and medical comorbidities, as shown in the screenshot in Fig. 1.

For consistency, it was agreed that for any specific condition/treatment, the same PROMs should be collected across the country, so national agreement was essential. We engaged with clinical groups across a range of conditions to identify areas where there was an appetite to set up PROMs collection with WG advising on priority areas. Before they can be considered for use on the programme, all tools must be agreed across Wales by the clinical reference group for that condition so that there is consistency in collection.

A WG requirement meant that the initial focus for collection was targeted at orthopaedics, cataracts, heart failure and lung cancer. There was strong clinical support and agreement on the choice of tools for a number of treatments within these fields, and a range of orthopaedic conditions were chosen as the first condition-specific tools for inclusion. Orthopaedic colleagues chose the Oxford Hip and Oxford Knee Scores [26] to mirror the collection by NHS England [27], and reflecting the requirements of organisations such as the National Joint Registry [28]. Other priority areas were identified as cataract surgery, heart failure and lung cancer, where condition-specific tools were also agreed. This was partly facilitated by the availability of ICHOM (International Consortium for Health Outcomes Measurement) standard sets which suggests PROMs in a range of conditions, and it was agreed that these were appropriate for use in Wales. A strategic partnership between NHS Wales and ICHOM supported practical implementation of lung cancer PROMs collection. Within clinical areas where there were no preferred tools, a standardised approach was developed to help identify potential options. This consisted of an initial review of available tools to identify appropriate options and review factors of the tools including the aim of the tool, whether they are validated for use in a UK population, target population and age, mode of collection, concepts of interest and domains. Other aspects for consideration are costs, ability to gain permission to use the tool electronically, and permission to carry out Welsh language translation and validation. Further detail on the PROMs selection process is available in Palmer et al.[29].

With respect to PREMs, a tool was required, which could be used across different settings so that one set of measures could be applicable to all clinical areas. However, no existing suitable tool was identified. Patient experience teams across Wales were consulted, and it was agreed that large-scale collection of standardised or “universal” experience measures that are applicable to all secondary healthcare settings would allow HBs to identify key areas to target local initiatives. A set of PREMs previously developed by WG [30] was identified; however, these were unsuitable for areas such as emergency care and had not been tested with patient groups. This PREMs set was adapted and validated as a core set for use on the platform, and covers topics such as timely assistance, involvement with decisions about care and Welsh language requirements [31].

### Integration with existing clinical systems

In order to realise the full potential of the PROMs system to add value, it needs to be fully integrated with existing clinical systems to reduce manual input and to allow responses to be linked to clinical data including patient admissions and outpatient appointments. The design includes automatic triggers around pre-agreed scheduled time points, e.g. every 6 months and following key events such as surgery. This provides flexibility and ensures that patients with the same condition are asked to complete the same surveys at the same time points to facilitate comparative analysis and provide standardised data for decision making. There are a number of patient administration systems across Wales, and the platform needs to link effectively with all of these to enable data to flow effectively.

Patient-identifiable PROMs will be collected on a national basis and will be stored in the NWIS data warehouse alongside other nationally held datasets that include information on admitted patient care and outpatient appointments. Linkage between these datasets will form the basis of health economic analysis by comparing costs for consumables, treatment options and the impact of different patient demographics.

## Results

Here, we present the results of our work to develop an electronic platform which uses recognised tools to collect patient-reported data in a form which facilitates both individual patient-level use and cohort analysis.

**Fig. 2:**
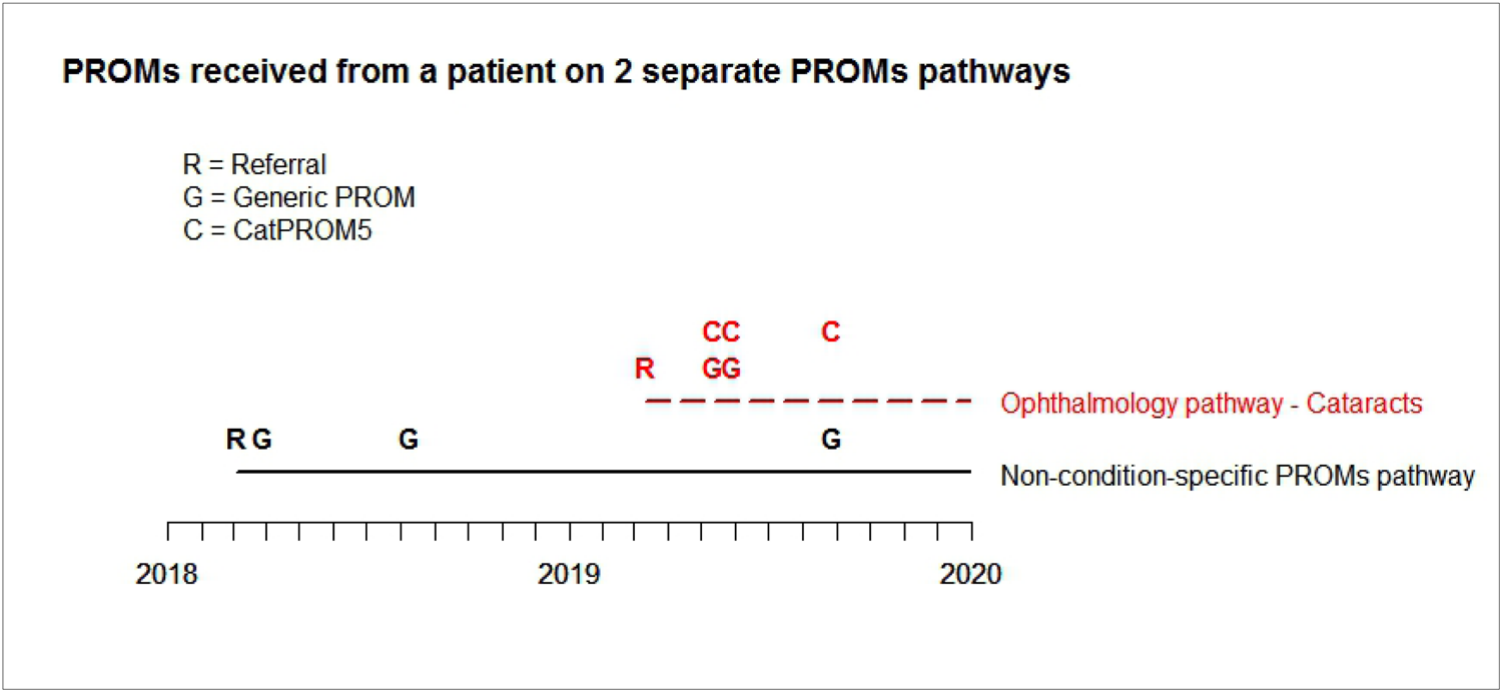
Shows a single patient referred onto a generic PROMs pathway in early 2018 and then referred onto a cataract pathway in early 2019. This patient would complete a generic PROM and a condition-specific PROM (in this case, CatPROM5) at the time points indicated

A fully integrated national electronic data collection platform has been successfully deployed for use in HBs and Trusts in Wales with the first data collected in June 2016. Uptake across HBs has varied as different clinical areas have implemented use at their own pace, with some organisations choosing to use alternative systems to meet specific needs. However, all but one health board in Wales are currently using the national electronic platform for PROMs collection in some capacity. Methods of collection vary with most HB’s collecting only for specific conditions such as lung cancer or orthopaedics, while Cardiff & Vale UHB opted to roll out large-scale collection whereby all patients are invited to complete the generic tool. This is collected alongside relevant condition-specific tools where available. An overview of current collection on the platform is available in Table 1. As detailed previously [21], two methods of collection currently exist, one designed so that patients can complete surveys on electronic devices in-clinic, and another “remote” system allowing collection on electronic devices in any setting. As the “remote” collection is connected to the HBs local patient administration system, programming within the system means that once patients are assigned to a relevant clinical pathway, e.g. arthroplasty, our collection system automatically invites them to complete a PROM for that condition at the scheduled time points. The patient acknowledgement letter includes an invitation to complete the PROM via a web-link with a unique identifier which enables them to log in. Once the PROM is completed, the data are stored in a national repository and a copy added to the Welsh Care Records Service for access by clinical teams. PROMs collection is embedded seamlessly throughout the patient journey, with the system automatically sending out further PROM invitations at predetermined time points. The system also allows ad-hoc completions with the invite to complete the PROM remaining open so that once invited onto the system patients can complete the PROM at any time and repeat this at will. This means they do not need to wait until a request is sent to them and can complete one as needed, e.g. during a clinic visit. This is particularly useful for conditions which may require regular clinic appointments at times during the treatment journey. Patients can be on more than one pathway at any time as illustrated in Fig. 2.

**Table 1:** Current status of PROMs collection using the National platform

Health board	Site	Clinical speciality	Pathway
Aneurin Bevan University Health Board		Not collecting	
Betsi Cadwaladr University Health Board	Central area (Conwy & Denbighshire)	Trauma & orthopaedic General surgery and colorectal	All Orthopaedic pathways Generic
	BCU East area: Wrexham Maelor hospital	Trauma & orthopaedic	All Orthopaedic pathways
	BCU West area: Ysbyty Gwynedd hospital	Trauma & orthopaedic	Hip arthroplasty Knee arthroplasty
Cardiff & Vale University Health Board	All sites	All specialities Ophthalmology Dermatology Cardiology Trauma & orthopaedic	Generic Cataract Dermatology Heart failure Shoulder arthroplasty Elbow arthroplasty Hip arthroplasty Hip non-arthroplasty Knee arthroplasty Knee non-arthroplasty Knee ACL Knee Osteotomy Hand Arthritis Hand Arthritis Non-Arthroplasty Hand—dupuytrens Hand—general (incl: non-wrist trauma) Hand—carpal tunnel Wrist—rheumatoid Wrist—general conditions Trauma (wrist/carpal injury)
Cwm Taf Morgannwg University Health Board	All sites	Trauma & orthopaedic	Hip arthroplasty Hip non-arthroplasty Knee arthroplasty Knee non-arthroplasty Knee ACL Knee osteotomy Patellofemoral conditions
Hywel Dda University Health Board	Withybush General Hospital	Respiratory	Lung cancer
	Bronglais General Hospital Withybush General Hospital Glangwili General Hospital	Trauma & orthopaedic	Hip arthroplasty Hip non-arthroplasty Knee arthroplasty Knee non-arthroplasty
Powys Teaching Health Board	All sites	Cardiology	Heart failure
Swansea Bay University Health Board	Morriston hospital	ENT Respiratory Cardiology Ophthalmology	Tonsillectomy Lung cancer Heart failure Cataract
	All sites	Trauma & orthopaedic	Hip arthroplasty Knee arthroplasty

NB: Swansea Bay UHB is the successor body to the former Abertawe Bro Morgannwg University Health Board following a change in name and boundary on 1 April 2019

Data are stored centrally at NWIS and available to individual HBs in order to maximise use on a patient level and population basis. The “in-clinic” system is not connected to the local patient administration systems, therefore, involves a member of the clinical team logging onto the system to select the correct tool for the patient to complete. As a result, PROMs data collected with this method are not added to the patient record; however, patient responses are still made available via a standalone clinical portal which allows clinicians to see the completed PROM.

While this collection method has less functionality and therefore increased limitations compared to the “remote” system, it is available as an alternative for clinics where remote collection is not yet available. It was originally developed to pilot the system, and intended as a temporary measure only. This system also has an increased risk of error due to the potential for the clinic staff to load the incorrect PROM or patient information. There is also an additional burden on clinic staff to facilitate this system, and it relies on local internet connections. As the “remote” collection extends, the “in-clinic” system will become obsolete and will be retired in the near future.

For both methods of collection, collected data are stored centrally at NWIS and available to all individual HBs in order to maximise use of their own data on a patient level and population basis, and for data linkage analysis. The dataset as a whole is available for national use.

### Progress and patient involvement

Over 30 PROMs tools have been agreed by the appropriate clinical groups and included on the system covering a range of clinical areas including orthopaedics, dermatology, heart failure and tonsillectomy as detailed in Table 2. Licences for these have been gained allowing the programme to collect in both English and Welsh. The Welsh Language (Wales) Measure [32] gives the Welsh language official status in Wales and encourages the equal provision of Welsh services to the public. To meet this requirement, patient-facing areas of the PROMs platform including the tools themselves are available in both English and Welsh. All PROMs have been translated and validated following the requirements of the licence or ISPOR (International Society for Pharmacoeconomics and Outcomes Research) guidelines [33]. As most PROMs have been developed on paper, the programme has converted paper formats to electronic format, following ISPOR guidelines for equivalence [34]. All Welsh language and electronic validation have involved patients’ input, with approximately 300 patients interviewed to date. A PREMs survey was developed and validated with patients and adopted for use across Wales [31]. Service users and key groups have been consulted in interviews and focus groups to gain feedback on the PPEP website [35]), to improve the FAQs and usability, and to identify how we can improve access to groups such as the visually impaired. The website interface has sections providing information about the programme and FAQs. A contact email allows patients to send questions to the team if any technical issues arise.

**Table 2:** Nationally agreed pathways and included tools

Sub-specialty	Pathway	Tools
Generic	PROM—generic (adults)	EQ-5D-5L, WPAI, ‘about you’
	PREM	NHS Wales PREM
Hip	Arthroplasty	OHS
	Non-arthroplasty	IHOT-12
Knee	Arthroplasty	OKS
	Non-arthroplasty	KOOS
	ACL	IKDC + KOOS + Tegner
	Osteotomy	KOOS + Oxford knee score + OKS-APQ
	Patellofemoral conditions	Kujala
Foot and ankle	Arthroplasty	MOxFQ
	Non-arthroplasty	MOxFQ
Shoulder	Arthroplasty	OSS
	Non-arthroplasty	OSS
	Instability	OSIS
Elbow	Arthroplasty	OES
	Non-arthroplasty	OES
Hand	Hand arthritis	PEM + Brief MHQ
	Hand arthritis Non-Arthroplasty	PEM + Brief MHQ
	Hand—dupuytrens	PEM + URAM
	Hand—general (incl: non-wrist trauma)	PEM + QuickDash
	Hand—carpal tunnel	PEM + Boston CTQ
	Wrist—rheumatoid	PEM + PRWHE + brief MHQ
	Wrist—general conditions	PEM + PRWHE
	Trauma (wrist /carpal injury)	PEM + PRWHE
ENT	Tonsillectomy	T-14
	Rhinosinusitis	SNOT-22
Ophthalmology	Cataract	CatPROM5 (original collection via CatQuest-9 s
Cancer	Lung cancer	EORTC QLQ-C30 + EORTC QLQ-LC13
	Neuro endocrine tumours (NETs)	EORTC QLQ-C30—EORTC GI.NET21 + Bristol stool chart
	Prostate cancer	EPIC 26
Dermatology	General dermatology (rashes)	DLQI
Cardiac	Heart failure	KCCQ-12 + PROMIS SF + PHQ-2

*Boston CTQ* Boston carpal tunnel syndrome questionnaire [36], *Brief MHQ* brief michigan hand questionnaire [37], CatPROM5 [38]; CatQuest-9S [39]; *DLQI* Dermatology Quality-of-Life Index [40], *EORTC QLQ-C30* The European Organization for Research and Treatment of Cancer Quality-of-Life Core Questionnaire 30 [41], *EORTC QLQ-GI.NET21* European Organization for research and treatment of cancer quality-of-life questionnaire—neuroendocrine carcinoid module [42], *EORTC QLQ-LC13* European organization for research and treatment of cancer quality-of-life questionnaire – lung cancer module [43]; *EPIC 26* Expanded Prostate Cancer Index Composite-26 [44], *EQ-5D-5L* EQ-5D- 5 level [23], *iHOT-12* 12-item International hip outcome tool [45], *IKDC* International knee documentation committee subjective knee evaluation form [46], *KCCQ-12* Kansas City cardiomyopathy questionnaire [47], *Kujala* Kujala anterior knee pain scale [48], *KOOS* knee injury and osteoarthritis outcome score [49], *MOxFQ* the manchester-oxford foot questionnaire [50], *myPOS* myeloma-specific palliative care outcome scale [51], *NHS Wales PREM* NHS Wales experience: patient-reported experience measure [52], *OES* The Oxford elbow score [53], *OHS* The Oxford hip score [54], *OKS* The Oxford knee score [55], *OKS—APQ* The Oxford knee score activity and participation questionnaire [56], *OSIS* The Oxford shoulder instability score [57], *OSS* The Oxford shoulder score [58], *PEM* patient evaluation measure [59], *HQ-2* patient health questionnaire [60], *PRWHE* patient-rated wrist/hand evaluation [61], *PROMIS SF* PROMIS scale v1.2—global health [62], *QuickDASH* the disability of the arm, shoulder and hand score [63], *SNOT-22* sino-nasal outcome test [64], *T-14* paediatric throat disorders outcome test, [65] = Tegner activity scale [66], *URAM* Unite´ Rhumatologique des Affections de la Main scale [67], *WPAI* work productivity and activity impairment questionnaire [25]

To date, over 60,000 patient submissions have been received comprising over 110,000 individual PROMs and PREMs.

### Utilisation of PROMs data

Patients are currently able to receive a copy of their response following completion, which can be printed. Their responses are automatically saved into their own patient record for clinical use on an individual basis, e.g. during the consultation to inform the clinical team, enhance the patient–clinician discussion and improve opportunities for shared decision making. This information allows the patient to monitor their own health as a snapshot and over a period of time and allows the clinician to understand aspects of health which are most problematic to the individual patient. This can highlight areas of health which might otherwise have gone unnoticed. It can also be used to manage patient expectations of the potential outcomes of treatment and support discussions on the impact of a healthy lifestyle.

Availability of PROMs to clinical teams is beginning to help drive discussions regarding service transformation, and the introduction of PROMs collection within orthopaedics in Cardiff & Vale UHB has reduced the need for low-value appointments by 70% [19]. Other preliminary work involves remotely reviewing patients using their PROM responses at six months post-surgery to determine if they require a hospital appointment. Early analysis from one organisation suggests that fewer patients than expected require a face-to-face visit, which could significantly reduce follow-up clinic pressure. Linked collated data are also in the early stages of being analysed for clinical groups and governmental use, with the exploration of the impact of lifestyle choices such as smoking status, alcohol use, BMI and exercise on health-related quality of life. Whilst these analyses are driving discussions on future improvements, helping to pose new questions and encouraging engagement with the programme, we recognise that such analysis needs a larger dataset to address issues such as confounders, missing data and bias. However, these kinds of questions fit well with the VBHC agenda and could illustrate how improved symptoms can be achieved effectively by health management initiatives such as weight loss. The PROMs data collected are still in its infancy and will need time to develop; however, as it matures, the availability of a national dataset has the potential to support quality improvement work [68], burden of illness studies [69], and comparative and cost-effectiveness research [70].

## Current status and moving forwards

The PPEP has recently been adopted into the national VBHC Programme in Wales within the Value in Health (ViH) team, to support its approach to delivering prudent healthcare and achieving the best possible healthcare outcomes for our population with the resources that we have [71]. As it extends across Wales, data collection and analysis support work to identify and reduce unwarranted variation in services and outcomes by using PROMs to pinpoint where variation occurs in different areas of care. These include variations in pre-operative health and outcomes of treatment. This knowledge can subsequently lead to positive service delivery changes and is illustrated by independent work carried out in ABUHB where the use of PROMs and activity-based costing was used to identify the treatment pathway that offered better outcomes at a lower cost within the memory assessment services [19]. Although the data we currently hold is too immature to be used in high-level decision making, we are able to start to use it on an individual patient level while it grows, allowing us to developing our understanding.

ABUHB is also collecting PROMs on a commercial system set up in parallel with the national platform, and this has offered flexibility and additional learning opportunities. Under VBHC and the ViH Programme, the national platform will be one of an array of patient communication systems with various uses, and there will continue to be a number of collection systems in place to offer choice to users. Importantly, data will be collected to a common standard and fed into a national data repository for linkage and analysis. The aim to collate this data on a national basis is supported by a recent WG Health Circular which requires all NHS Wales health boards and trusts to consistently submit clinical audit and PROMs data, and to support the flow of this data to NWIS for national use [72].

In order to utilise the data collected under the umbrella of ViH, a range of stakeholder groups continue to be involved in the evolving analytical plan. As well as analytical teams within individual health boards, there is ongoing input from the ViH in-house analytical team, colleagues from the Finance Delivery Unit (FDU) and both technical and data visualisation teams within NWIS. These teams are able to bring a range of skills and experience to the programme which will allow us to consider and deal with ongoing complications including data quality, mixed modes of administration, goals, case-mix adjustment, data linkage and standardised rules and guidelines for analysis [29]. We continue to work with clinical and analytical teams across Wales to ensure that we are able to identify priority areas and produce meaningful outputs.

**Fig. 3:**
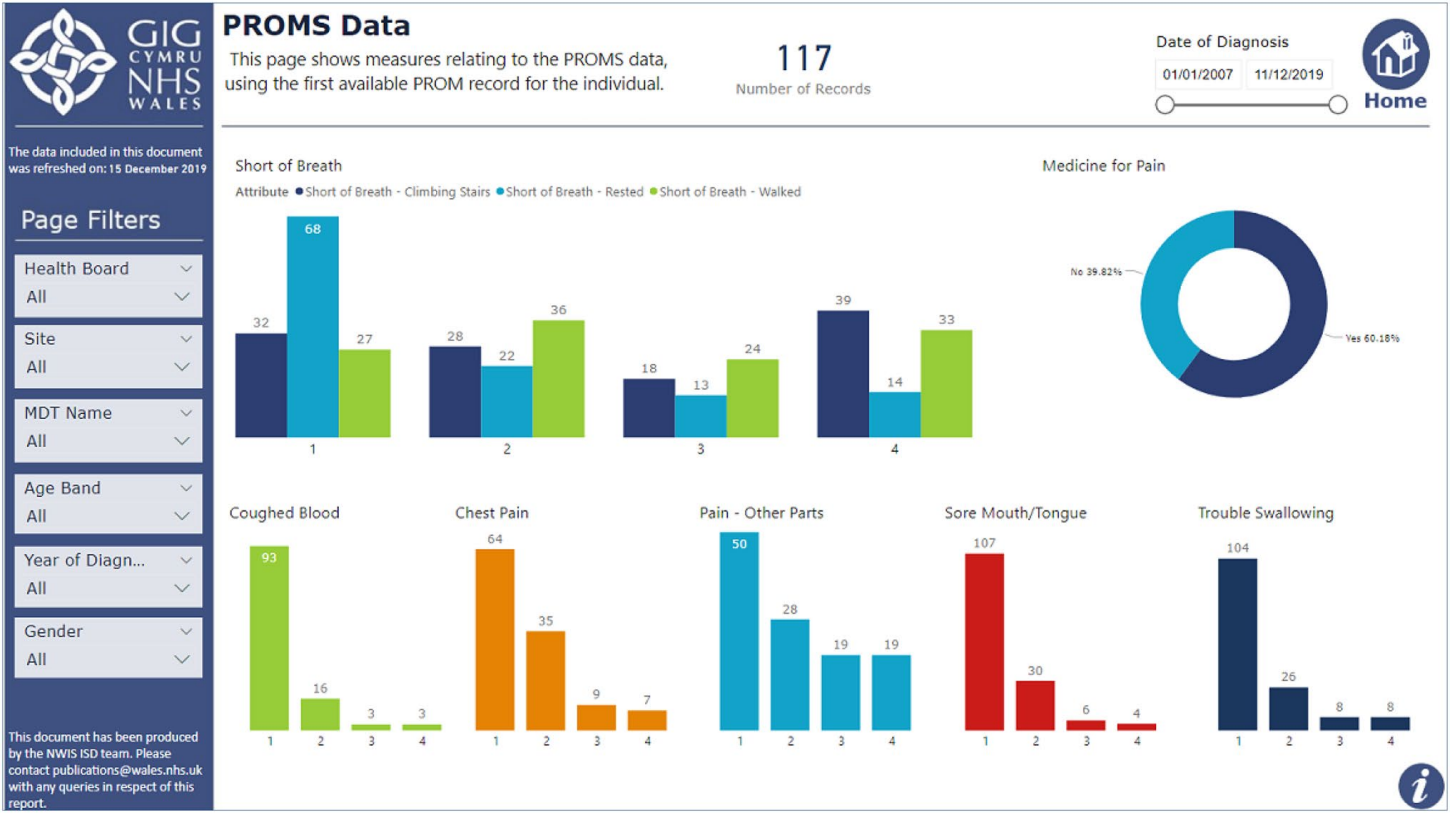
Shows a screenshot of one of the data dashboards available to view

The national platform continues to improve, and current developments within ViH focuses on data visualisation to support shared, informed decision making. PROMs responses available within the patient record will allow clinicians to visualise an individual’s PROMs responses over time and highlight where symptoms or problems are improving or worsening using data visualisations such as Red, Amber, Green alerts and arrow systems. These can allow clinicians to easily see how symptoms are responding to treatment and help identify changes in aspects of health. As well as allowing clinicians to monitor changes, it also provides a clear visual tool to support one-to-one discussions and shared decision making with patients in the clinic environment. This work is being piloted in lung cancer and will allow clinical teams to closely monitor patient symptoms to help focus the clinic appointment on the most relevant discussions and treatment decisions. Data dashboards are also being finalised which allow clinical teams to visualise the collated data available for specific conditions so that they can easily assess groups of patients at different stages of care and compare variables such as gender, stage of disease (if applicable), health board and age. This work has involved close collaboration between technical teams and clinicians from each speciality in order to produce a bespoke output for each specialism. A screenshot of the prototype of one data dashboard is available as an example in Fig. 3. As we aim to increase the use of the system to support an improvement in response rates, the use of a text reminder system has been piloted. This can send out automated messages to patients reminding them to complete a PROM before their appointment and is being tested to assess the effect on response rates.

We continue to improve the usability of the system and dataset through a constant process of review and refinement. Work is planned to improve the patients’ view of their own data so they can also view longitudinal completions.

## Discussion

The programme has overcome a range of technical and practical difficulties throughout implementation and is functioning as intended, integrating with other clinical platforms. The use of PROMs during the clinic appointment is putting patients at the centre of care by ensuring that they are fully involved in consultation discussions and decision-making processes. Imminent developments such as the introduction of data visualisation tools will make it easier to utilise and illustrate data in an accessible and meaningful way. Evidence suggests that sharing data derived from PROMs collection can improve patient engagement, encouraging patients to be more engaged and participate more actively in their healthcare [22].

## Current weaknesses

The “in-clinic” collection system is still in use in some areas and lacks some of the functionality of the remote system. Ongoing work to maximise the availability of the remote collection system will allow teams to migrate over to this system so that the in-clinic system can be retired. Currently, generic PROMs data collection has dominated, and despite its growing use, pre- and post-treatment condition-specific data are limited. To facilitate case-mix and cohort analysis for decision making, it is important to have larger datasets which take time to accumulate. This may delay use of the data for some purposes while the data mature. In addition, the data are complex and are available to a number of end users; therefore, it is important that methodologies are developed to facilitate reproducible reporting and that reporting methods are transparent and consistent to prevent discrepancies between organisations.

There are remaining difficulties in accurately determining response rates due to a number of complex factors [29], but it is estimated that our response rates are currently low while the platform has been overcoming initial developmental challenges. This has allowed us to progress the platform to its current status without raising expectations prematurely. Now that it is widely available we will increase patient and clinician outreach to improve response rates. Electronic data collection may disadvantage some users [73], with a 2018 realist synthesis identifying a “digital divide”, leading to inequities in the ability to access and gain from digital innovation [74]. While the reasons for this are complex, factors include education, income and generational status as well as chronological age. Similarly, findings from a recent UK study suggest that older people and those with a long-standing illnesses or disabilities are less likely to have internet access [75] which may be a barrier to their ability to interact with the system. Further work is required to understand the factors leading to potential digital inequalities so that steps can be taken to mitigate against them.

As noted, there has been uneven implementation across the HBs with some collecting across all conditions, some in specific specialities and one currently not using the national platform at all but using an alternative commercial system. A number of HBs are using the national platform and commercial systems in tandem. This has led to additional complexities in collecting data into a single repository both from technical and data protection viewpoints. The numerous different patient administration systems (even within a single health board), levels of engagement, and requirements for additional functionality have been among the barriers to a more comprehensive roll out. However, the different options for collection have allowed health boards to develop their systems in a way that best suits their own needs, priorities and technical requirements. We have focused initial work in areas where clinical staff has been most engaged to allow us to test functionality, identify issues within smaller scale pilots and showcase outputs. The national system is available across all Wales for HBs to use if they choose, and its development has, thus, met the original aims of the programme. We are continuing to work with clinical and technical teams to improve functionality and clinical interest. As uptake spreads, additional electronic collection platforms such as commercially available systems and additional PROMs tools will become available which over time will allow groups to have a flexible approach and support growth of national collection.

## Strengths and benefits

The use of an electronic system keeps costs low as no manual data input is required. This also reduces the risk of human error and time spent on data cleaning [76], while providing instantaneous feedback. The nationalisation of collection has also reduced costs and burdens related to PROM licensing as we have been able to negotiate licences to cover collection across a large geographic area and prevented the need for each user (e.g. health board) to obtain individual licences. With the expertise of the teams involved, we have been able to translate and validate most tools in-house. With recent quotations from external providers to translate and validate a single tool for in the region of $7,000, this provides a significant cost saving. Teams within the programme have provided expert support in the technical development and implementation of the platform, and there is capacity to develop targeted reports for clinical teams and national audiences.

Although electronic PROMs capture has potential drawbacks regarding accessibility, data show that use amongst older people, for example, has increased substantially in recent years [73]. In order to help reduce access issues and to improve usability, our system allows a third party, such as a family member, friend, carer or clinical staff to assist with completion. We have also supported a number of clinics in purchasing electronic tablets to use in hospital waiting rooms so patients can complete PROMs while they wait for their appointment. In some areas, this is being further supported by volunteers to help patients who require assistance. It is likely that with time, internet access and IT literacy will increase which may improve our collection rate. We also plan to provide access to paper PROMs in some areas to improve collection rates.

Clinicians and healthcare providers worldwide are encouraged to collect patient data with groups such as ICHOM developing standard sets to facilitate collection and comparison [77]. The practicalities of this can be challenging for busy clinical teams with already limited resources. The national programme has allowed clinical groups in Wales to implement PROMs collection with minimal input, and feedback from clinical groups has been positive with patients also providing encouraging feedback on the process.

Traditional PROMs collection such as the NHS England National PROMs programme has been remote from direct patient care [78]. Our system is directly linked to patient care as data are easily accessible to the patient and their clinician through platforms such as the Welsh Care Records Service and NWIS’ Clinician’s Portal. This can prove beneficial in the clinic environment [79]. As well as providing an additional use of the data, this helps make PROMs collection more relevant, which supports change management.

## Learning

We have proven that Despite challenging, it is feasible to incorporate electronic PROMs collection across organisations with different clinical systems, and to introduce their use to inform daily clinical care and decision making. For example, findings from the data on differences in outcomes by implant brand will allow colleagues in NHS Wales to make informed decisions during their procurement processes. As the data grow, we will be able to identify which healthcare services offered in Wales provide the best outcomes based on data provided by patients themselves. We are collecting data from a large number of patients across a broad range of clinical conditions. For some of these specialisms, PROMs use in clinical care is novel, and our work throughout the country is providing us with an insight into how our patients feel in a way that has not yet been fully investigated outside the research arena.

The amalgamation of different collection platforms under VBHC has allowed us to share knowledge and experiences across Wales. The development of data visualisation tools and data dashboards, plus the use of virtual reviews illustrates how PROMs collection can be an effective method of enhancing communication between the clinician and patient, both within and outside the clinic setting. We have involved clinical teams throughout the development of our programme including on the reporting and data visualisation work. There is currently significant interest from staff who are keen to access the PROMs data to inform their work. The use of virtual reviews is spreading which has the potential for additional clinics to prioritise clinic appointments and to free up clinic space for those in most need. As we start to resume routine care in the NHS following the Covid-19 outbreak, the use of remote methods of assessing health has become even more beneficial to prioritise care and reduce the need for unnecessary hospital visits. As we continue to embed PROMs into direct patient care, we will work with clinical teams and patients to raise the profile of the national PROMs platform, to improve the response rate and increase awareness of the potential of the system to improve patient care. As there are a number of clinical systems across Wales, setting central triggers for PROMs collection is challenging and may not be the most appropriate way forward if we wish to remodel care. Our experience to date suggests that communications should be locally facilitated but driven according to nationally agreed schedules to ensure that data are comparable whilst providing flexibility to fit local requirements and systems.

Perhaps most importantly, we have learned that patients want to be able to track their own outcomes and see the progress they are making over time. This would appear to be one method of involving patients in the process and providing an incentive to complete their PROMs. Data visualisation work will provide further opportunity to continue to improve communications with patients through involving patients in design processes to ensure that data are being presented in an accessible and engaging format. Work which allows patients to measure their own health and health changes against other similar patient cohorts may also help to improve engagement. Our ongoing work will gather patient feedback on their experience of this process.

## Abbreviations

ABUHB: Aneurin Bevan University Health Board
BMI: Body Mass Index
FAQ: Frequently asked questions
HB: Health board
HRQoL: Health-related quality of life
ICHOM: International consortium for health outcomes measurement
ISPOR: International society for pharmacoeconomics and outcomes research
IT: Information technology
NHS: National health service
NWIS: NHS Wales informatics service
PPEP: PROMs, PREMs and effectiveness programme
PREMs: Patient-reported experience measures
PROMs: Patient-reported outcome measures
UHB: University health board
UK: United Kingdom
US: United States
VBHC: Value-based healthcare
ViH: Value in health
WG: Welsh government
WPAI: Work, Productivity and Activity Index
